# A Perturbation Model of Gradient Energy Anisotropy for Phase-Field Simulation of Ferroelectrics

**DOI:** 10.3390/ma19071445

**Published:** 2026-04-04

**Authors:** Xiaoming Shi, Jiecheng Liu, Ke Xu, Haoyu Wang, Zheng Wang, Nan Wang, Houbing Huang, Zhuhong Liu

**Affiliations:** 1 Department of Physics, University of Science and Technology Beijing, Beijing 100083, China; 2 School of Interdisciplinary Science, Beijing Institute of Technology, Beijing 100081, China; xuke@bit.edu.cn; 3 School of Science, Guilin University of Aerospace Technology, Guilin 541004, China; 4 Department of Materials Science and Engineering, Guangdong Technion-Israel Institute of Technology, Shantou 515063, China

**Keywords:** perturbation model, anisotropic gradient tensor, antiferroelectric

## Abstract

The efficient and accurate description of gradient energy anisotropy remains a significant challenge in the phase-field modeling of ferroelectric/antiferroelectric (FE/AFE) composite systems. To address this limitation, we have developed a perturbation model for solving anisotropic gradient energy based on Fourier spectral methods. Through a Fourier-space perturbation scheme, we achieve the ability to treat the full anisotropic gradient energy tensor with spatial variations, overcoming limitations of previous constant-coefficient or isotropic approximations. The application of this model to FE/AFE composites demonstrates exceptional robustness and convergence efficiency. Numerical results indicate that the proposed perturbation scheme can accurately reproduce antiferroelectric phase diagrams and AFE-FE phase transition pathways under varying gradient energy parameters. Furthermore, the algorithm exhibits superior scalability, allowing for a seamless extension to three-dimensional (3D) simulation domains. This capability facilitates the visualization of complex nanodomain structures and reveals the intricate 3D evolution mechanisms of polarization textures.

## 1. Introduction

The rapid advancement of high-power pulsed-power technologies has imposed increasingly stringent demands on dielectric materials, necessitating superior energy density, charge–discharge efficiency, and response speed [1,2,3,4]. Consequently, relaxor ferroelectrics (RFEs) and antiferroelectrics (AFEs) have emerged as cutting-edge candidates for advanced dielectrics [5,6]. These materials exhibit exceptional energy storage capabilities due to their unique polarization response mechanisms. Specifically, RFEs are characterized by diffuse phase transitions, frequency-dispersive dielectric responses, and the presence of polar nanoregions (PNRs), which collectively result in slender hysteresis loops, high energy density, and low loss [7,8,9]. Conversely, AFEs can undergo a field-induced phase transition between antiferroelectric and ferroelectric states, enabling rapid polarization switching and yielding high recoverable energy density along with excellent cycling stability [10]. These attributes render them highly promising for applications in high-energy-density capacitors and pulsed-power systems.

The fundamental physical mechanism governing the performance of RFEs and AFEs lies in their intricate local heterogeneous nanostructures. Features at the nanoscale—such as PNRs, chemical order–disorder distributions, and lattice distortions—directly modulate the polarization behavior and phase transition kinetics [11]. Therefore, a profound theoretical understanding and precise description of the formation and evolution of these local structures are pivotal to unraveling the origins of their superior performance. Current theoretical modeling often employs phase-field simulations based on fluctuations induced by chemical doping, using disorder fields to mimic dopant effects on phase behavior. However, existing models predominantly focus on phase fluctuations, elastic energy fluctuations, and electric field energy fluctuations [12,13], while largely neglecting the critical role of gradient energy fluctuations. This simplification presents a significant theoretical deficiency, particularly in complex systems where ferroelectric and antiferroelectric phases coexist or compete. Due to the cooperative tilting of oxygen octahedra and lattice strain, the spatial variation in polarization exhibits a strong directional dependence, leading to a pronounced anisotropy in the gradient energy. Ignoring this effect hinders the accurate capture of non-uniform polarization evolution, thereby compromising the precise prediction of phase transition pathways, domain structure evolution, and energy storage mechanisms.

To accurately describe such anisotropic effects, it is imperative to develop a phase-field theory that incorporates gradient energy anisotropy [12,14]. In terms of numerical solution strategies, the Fourier spectral method has become a mainstream approach for solving phase-field equations owing to its high efficiency under periodic boundary conditions. By directly computing spatial differential operators in the frequency domain, this method exhibits spectral convergence, enabling larger time steps and significantly enhanced computational efficiency compared to real-space finite difference methods. Particularly for material systems exhibiting anisotropy in elasticity, dielectricity, thermal conductivity, or electrical conductivity [15,16,17,18,19,20], the perturbation Fourier-space iterative scheme often converges to a stable solution within only a few iterations, demonstrating good computational robustness. Moreover, the perturbation method can also be employed to solve ultrafast phase-field kinetic equations [21].

Nevertheless, the prevailing Fourier-space algorithmic frameworks either neglect the spatial heterogeneity of the gradient energy coefficients, or they rely on real-space discretizations that do not fully exploit the efficiency of the Fourier spectral method. Specifically, none of the existing spectral implementations can simultaneously handle a full anisotropic tensor of gradient coefficients that also varies in space. This algorithmic gap severely limits high-fidelity simulations of polarization texture evolution, multi-phase competition, and field-response mechanisms in complex ferroelectric–antiferroelectric coexisting systems. To address this critical scientific challenge, this study proposes a Fourier-space perturbation model for solving anisotropic gradient energy in ferroelectric materials. By constructing an anisotropic gradient energy operator in reciprocal space, our model fully accounts for the asymmetric response of polarization gradients across different crystallographic orientations, thereby achieving accurate discretization and an efficient solution of anisotropic gradient energy. The development of this model not only remedies the theoretical shortcomings of existing phase-field simulations but also provides a powerful computational tool for deeply understanding of the nanostructure–property relationships in relaxor ferroelectric/antiferroelectric materials, paving the way for the rational design of next-generation high-performance energy storage materials.

## 2. Theory Model

In the global coordinate system, a global polarization *P* and a displacement field *u* were adopted as the order parameters in the phase-field model. The temporal evolution of the polarization is described by the time-dependent Ginzburg–Landau (TDGL) equation and the stress/electric field equilibrium equation [22]:

(1) $$\begin{array}{c} \frac{\partial P_{i}}{\partial t}=-L\frac{\delta F}{\delta P_{i}}+E_{i}^{\mathrm{thermal}\;}\\ \frac{\partial }{\partial x_{j}}\left(\sigma_{ij}(r,t)\right)=0\\ \nabla \cdot D=\rho_{f} \end{array}$$

Here, *L* is a kinetic coefficient related to domain wall mobility; *F* is the total free energy of the system; $\frac{\delta F}{\delta P_{i}}$ is the thermodynamic driving force given by the variational derivative of the total free energy with respect to the polarization, which governs the temporal evolution of the polarization field toward the free-energy minimum; $\sigma_{ij}$ is the stress tensor; *D* is the electric displacement; $\rho_{f}$ is the free charge density; and **r** and *t* are the spatial coordinate and time, respectively. $E_{i}^{\mathrm{thermal}\;}$ is the thermal field, which follows [23] $\left\langle E^{\mathrm{thermal}\;}\left(r_{1},t_{1}\right)\cdot E^{\mathrm{themal}\;}\left(r_{2},t_{2}\right)\right\rangle =2k_{B}TL\delta \left(r_{1}-r_{2}\right)\delta \left(t_{1}-t_{2}\right)$, where $k_{B}$ is the Boltzmann constant and T is the thermodynamic temperature. The total free energy of a bulk system can be defined as follows:

(2) $$F=F_{\mathrm{Land}\;}\left(P\right)+F_{\mathrm{grad}\;}\left(P\right)+F_{\mathrm{elastic}\;}\left(P\right)+F_{\mathrm{elec}\;}\left(P,E\right)=\int_{V}\left(f_{\mathrm{Land}\;}+f_{\mathrm{grad}\;}+f_{\mathrm{elastic}\;}+f_{\mathrm{elec}\;}\right)dV$$

Here, *F* includes the bulk free energy $F_{\mathrm{Land}\;}(P)$, domain-wall energy $F_{\mathrm{grad}\;}(P)$, elastic energy $F_{\mathrm{elastic}\;}(P)$, and electrostatic energy $F_{\mathrm{elec}\;}(P,E)$, where ***E*** is the applied static electric field. $f_{\mathrm{Land}}$, $f_{\mathrm{grad}}$, $f_{\mathrm{elastic}}$ and $f_{\mathrm{elec}}$ are corresponding energy densities.

The bulk free energy density can be expanded in terms of polarization components:

(3) $$\begin{array}{c} f_{Land}=a_{1}\left(P_{1}^{2}+P_{2}^{2}+P_{3}^{2}\right)+a_{11}\left(P_{1}^{4}+P_{2}^{4}+P_{3}^{4}\right)+a_{12}\left(P_{1}^{2}P_{2}^{2}+P_{1}^{2}P_{3}^{2}+P_{2}^{2}P_{3}^{2}\right)+a_{111}\left(P_{1}^{6}+P_{2}^{6}+P_{3}^{6}\right)\\ +a_{112}\left[P_{1}^{4}\left(P_{2}^{2}+P_{3}^{2}\right)+P_{3}^{4}\left(P_{2}^{2}+P_{1}^{2}\right)+P_{2}^{4}\left(P_{3}^{2}+P_{1}^{2}\right)\right]+a_{123}P_{1}^{2}P_{2}^{2}P_{3}^{2}+a_{1111}\left(P_{1}^{8}+P_{2}^{8}+P_{3}^{8}\right)\\ +a_{1112}\left[P_{1}^{6}\left(P_{2}^{2}+P_{3}^{2}\right)+P_{3}^{6}\left(P_{2}^{2}+P_{1}^{2}\right)+P_{2}^{6}\left(P_{3}^{2}+P_{1}^{2}\right)\right]+a_{1122}\left(P_{1}^{4}P_{2}^{4}+P_{1}^{4}P_{3}^{4}+P_{2}^{4}P_{3}^{4}\right)\\ +a_{1123}\left(P_{1}^{4}P_{2}^{2}P_{3}^{2}+P_{1}^{2}P_{2}^{4}P_{3}^{2}+P_{1}^{2}P_{2}^{2}P_{3}^{4}\right) \end{array}$$

where $a_{1}-a_{1123}$ are the Landau coefficients, by using higher-order Landau energy, different anisotropic free energy minima can be achieved to represent distinct ferroelectric phases. The elastic energy density can be described as follows:

(4) $$f_{\mathrm{elastic}\;}=\frac{1}{2}c_{ijkl}e_{ij}e_{kl}=\frac{1}{2}c_{ijkl}\left(\varepsilon_{ij}-\varepsilon_{ij}^{0}\right)\left(\varepsilon_{kl}-\varepsilon_{kl}^{0}\right)$$

where $c_{ijkl}$ is the stiffness tensor, $e_{ij}$ is the elastic strain tensor, $\varepsilon_{ij}$ is the total strain tensor, and $\varepsilon_{ij}^{0}$ is the eigenstrain

(5) $$\varepsilon_{ij}^{0}=Q_{ijkl}P_{k}P_{l}$$

where $\varepsilon_{ij}^{0}$ is the polarization-induced eigenstrain, and $Q_{ijkl}$ is the electrostrictive coefficient tensor. The electrostatic energy density $f_{\mathrm{elec}}$ of the system is given by

(6) $$f_{elec}=-P_{i}{\left(r\right)(E}_{i}\left(r\right)+E_{RF}(r))-\frac{1}{2}P_{i}(r)E_{i}^{in}(r)$$

where $E_{i}^{in}(r)$ is the E-field induced by the dipole moments, $E_{i}(r)$ is the applied electric field and $E_{RF}$ is the local electric field caused by random point defects.

The gradient energy density is given by

(7) $$\begin{array}{c} f_{G}\left(P_{i,j}\right)=\frac{1}{2}G_{11}\left(P_{1,1}^{2}+P_{2,2}^{2}+P_{3,3}^{2}\right)+G_{12}\left(P_{1,1}P_{2,2}+P_{2,2}P_{3,3}+P_{1,1}P_{3,3}\right)\\ +\frac{1}{2}G_{44}\left[{\left(P_{1,2}+P_{2,1}\right)}^{2}+{\left(P_{2,3}+P_{3,2}\right)}^{2}+{\left(P_{1,3}+P_{3,1}\right)}^{2}\right]\\ +\frac{1}{2}G_{44}^{\prime }\left[{\left(P_{1,2}-P_{2,1}\right)}^{2}+{\left(P_{2,3}-P_{3,2}\right)}^{2}+{\left(P_{1,3}-P_{3,1}\right)}^{2}\right] \end{array}$$

where $P_{i,j}=\frac{\partial P_{i}}{\partial x_{j}}$; $G_{11},G_{12},G_{44}$ and $G_{44}^{\prime }$ are the gradient energy coefficients.

Thus, the total gradient energy is given by [5]

(8) $$F_{G}=\int_{V}f_{G}\left(P_{i,j}\right)dV$$

By performing the variational derivative of the polarization gradient energy, we obtain

(9) $$\begin{array}{c} \frac{\delta F_{G}}{\delta P_{1}}=-G_{11}\frac{\partial^{2}P_{1}}{\partial x_{1}^{2}}-G_{12}\left(\frac{\partial^{2}P_{2}}{\partial x_{1}\partial x_{2}}+\frac{\partial^{2}P_{3}}{\partial x_{1}\partial x_{3}}\right)\\ -G_{44}\left(\frac{\partial^{2}P_{1}}{\partial x_{2}^{2}}+\frac{\partial^{2}P_{2}}{\partial x_{1}\partial x_{2}}+\frac{\partial^{2}P_{1}}{\partial x_{3}^{2}}+\frac{\partial^{2}P_{3}}{\partial x_{1}\partial x_{3}}\right)\\ -G_{44}^{\prime }\left(\frac{\partial^{2}P_{1}}{\partial x_{2}^{2}}-\frac{\partial^{2}P_{2}}{\partial x_{1}\partial x_{2}}+\frac{\partial^{2}P_{1}}{\partial x_{3}^{2}}-\frac{\partial^{2}P_{3}}{\partial x_{1}\partial x_{3}}\right) \end{array}$$

Next, we consider the more general case where the gradient energy is spatially anisotropic.

(10) $$F_{G}=\int_{V}f_{G}\left(P_{i,j},r\right)dV$$

where the gradient tensors are spatially anisotropic, by performing the variational derivative of the polarization gradient energy, we obtain Equation (S1). Here, we consider the common special case where the gradient energy coefficients satisfy $G_{12}=0$ and $G_{44}=G_{44}^{\prime }$. Thus, we have

(11) $$f_{G}=\frac{1}{2}G_{11}(r)\left(P_{1,1}^{2}+P_{2,2}^{2}+P_{3,3}^{2}\right)+G_{44}(r)\left(P_{1,2}^{2}+P_{2,1}^{2}+P_{2,3}^{2}+P_{3,2}^{2}+P_{1,3}^{2}+P_{3,1}^{2}\right)$$

The driving force of gradient energy density can be simplified as follows:

(12) $$\begin{array}{cc} \frac{\delta F_{G}}{\delta P_{1}}= & -G_{11}\frac{\partial^{2}P_{1}}{\partial x_{1}^{2}}-2G_{44}\left(\frac{\partial^{2}P_{1}}{\partial x_{2}^{2}}+\frac{\partial^{2}P_{1}}{\partial x_{3}^{2}}\right)\\ & -\frac{\partial G_{11}}{\partial x_{1}}\frac{\partial P_{1}}{\partial x_{1}}-2\frac{\partial G_{44}}{\partial x_{2}}\frac{\partial P_{1}}{\partial x_{2}}-2\frac{\partial G_{44}}{\partial x_{3}}\frac{\partial P_{1}}{\partial x_{3}} \end{array}$$

To ensure convergence of the iteration, we aim to solve the equation in Fourier space. However, due to the anisotropy of the gradient energy, it cannot be directly represented by multiplying the polarization in Fourier space by the wave vector. To address this, we propose a perturbation method in Fourier space for the solution. According to the convolution theorem $F[f(x)g(x)]=\hat{f}(k)*\hat{g}(k)$, we can obtain:

(13) $$\begin{array}{c} F\left[G(r)\nabla^{2}P(r)\right]=\hat{G}(k)*\left(-|k{|}^{2}\hat{P}(k)\right)\\ F[\nabla G(r)\cdot \nabla P(r)]=(ik\hat{G}(k))*(ik\hat{P}(k)) \end{array}$$

It can be seen that the variational derivative of the gradient energy can be expressed as a convolution in Fourier space. Thus, we decompose the gradient energy variational operator (L) into two parts: one is a constant term using average value ($L_{0}=\bar{G}\nabla^{2}$), and the other is a spatial perturbation term ($L-L_{0}$).

(14) $$L=L_{0}+\left(L-L_{0}\right)$$

Now, the TDGL equation in Fourier space is given by

(15) $$\frac{{\hat{P}}_{i}^{\left(n+1\right)}-{\hat{P}}_{i}^{\left(n\right)}}{\Delta t}=-L\left({\hat{N}}^{\left(n\right)}+\hat{L}P_{i}^{\left(n\right)}\right)$$

where ${\hat{N}}^{\left(n\right)}$ is the variational derivative of the total free energy from Landau, elastic and electric energy. By taking the Fourier transform of the gradient energy, one can obtain

(16) $${\hat{P}}_{i}^{\left(n+1\right)}=\frac{{\hat{P}}_{i}^{\left(n\right)}-L\Delta t\left({\hat{N}}^{\left(n\right)}+{\hat{R}}^{\left(n\right)}\right)}{1+L\Delta tk_{eff}^{2}}$$

where $k_{eff}^{2}={\bar{G}}_{11}k_{x}^{2}+2{\bar{G}}_{44}\left(k_{y}^{2}+k_{z}^{2}\right)$ and ${\hat{R}}^{\left(n\right)}=F\left[LP_{i}^{\left(n\right)}\right]-k_{eff}^{2}{\hat{P}}_{i}^{\left(n\right)}$.

## 3. Higher-Order Anisotropic Gradient Energy

In the antiferroelectric model, the second-order gradient energy coefficient is negative; therefore, higher-order gradient energy terms are required to stabilize the periodic antiferroelectric structure. Generally, the higher-order gradient energy can be expressed as follows:

(17) $$F_{grad}^{\left(2\right)}=\int_{\Omega }\frac{1}{2}H_{ijkl}\frac{\partial^{2}P_{i}}{\partial x_{j}\partial x_{k}}\frac{\partial^{2}P_{l}}{\partial x_{j}\partial x_{k}}dr$$

or we can use the simplified formation:

(18) $$F_{grad}^{\left(2\right)}=\int_{\Omega }\frac{1}{2}H\sum_{i=1}^{3}{\left(\nabla^{2}P_{i}\right)}^{2}dr$$

where H is the fourth-order gradient coefficients. For the system with inhomogeneous gradient coefficients, the driving force can be obtained:

(19) $$\frac{\delta F_{grad}^{\left(2\right)}}{\delta P_{i}}=\nabla^{2}\left(H(r)\nabla^{2}P_{i}\right)$$

Further expansion yields

(20) $$\begin{array}{cc} \frac{\delta F_{grad}^{\left(2\right)}}{\delta P_{i}}= & H(r)\nabla^{4}P_{i}+2\nabla H(r)\cdot \nabla \left(\nabla^{2}P_{i}\right)\\ & +\nabla^{2}H(r)\nabla^{2}P_{i} \end{array}$$

If solved in Fourier space, we obtain the following form:

(21) $$\begin{array}{c} F\left[H\nabla^{4}P\right]=\hat{H}(k)*\left(\left|k{|}^{4}\hat{P}(k\right)\right)\\ F\left[\nabla H\cdot \nabla \left(\nabla^{2}P\right)\right]=(ik\hat{H}(k))*\left(-ik|k{|}^{2}\hat{P}(k)\right)\\ F\left[\left(\nabla^{2}H\right)\nabla^{2}P\right]=\left(-|k{|}^{2}\hat{H}(k)\right)*\left(-|k{|}^{2}\hat{P}(k)\right) \end{array}$$

which clearly involves a convolution. Therefore, a perturbation method can be employed for improvement.

(22) $$\frac{{\hat{P}}_{i}^{\left(n+1\right)}-{\hat{P}}_{i}^{\left(n\right)}}{\Delta t}=-L\left({\hat{N}}^{\left(n\right)}+\bar{H}|k{|}^{4}{\hat{P}}_{i}^{\left(n+1\right)}+{\hat{R}}^{\left(n\right)}\right)$$

where $\bar{H}$ is the homogeneous part and $\hat{R}$ is the perturbation part; thus, we obtain

(23) $${\hat{P}}_{i}^{\left(n+1\right)}=\frac{{\hat{P}}_{i}^{\left(n\right)}-L\Delta t\left({\hat{N}}^{\left(n\right)}+{\hat{R}}^{\left(n\right)}\right)}{1+L\Delta t\bar{H}|k{|}^{4}}$$

where ${\hat{R}}^{\left(n\right)}=F\left[\delta F_{grad}^{\left(2\right)}/\delta P_{i}^{\left(n\right)}\right]-\bar{H}|k{|}^{4}{\hat{P}}_{i}^{\left(n\right)}$.

## 4. Results and Discussion

In our simulation, we employed the thermodynamic potential function of PZO material [5]. For the 2D simulation, we used a grid of 128Δx × 128Δy × 1Δz with Δx = Δy = Δz = 1.0 nm. To verify the stability and convergence of the algorithm, we computed the evolution of the total free energy for the FE/AFE composite system (with spatially varying gradient coefficients) using two different time steps: Δt = 0.02 and Δt = 0.1 (in normalized units). The results are shown in Figure S1. For both time steps, the total free energy decreases monotonically over time and eventually converges to the same stable equilibrium value. This confirms that the semi-implicit perturbation scheme preserves the thermodynamic consistency of the time-dependent Ginzburg–Landau equation. The convergence rate is faster for Δt = 0.1 than for Δt = 0.02, indicating that the algorithm remains stable even with a relatively large time step. To validate the domain structures, we examined the gradient energy anisotropy present throughout the system, which encompasses two main components. The first is the ferroelectric phase gradient energy anisotropy, where adjacent polarizations tend to align in parallel, albeit with distinct gradient energy coefficients. The second is the ferroelectric/antiferroelectric anisotropy, characterized by the coexistence of parallel and antiparallel polarization alignments in specific spatial regions.

**Figure 1:**
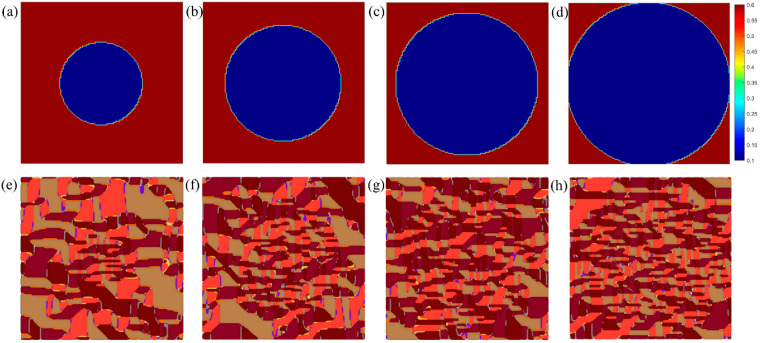
$G_{11}$ distribution and domain structure. (**a**–**d**) Circular ferroelectric anisotropy with low-gradient-energy regions ($G_{11}=0.1$) occupying 20%, 40%, 60%, and 80% of the total area, respectively. (**e**–**h**) The corresponding domain structures (Different colors represent different domain structures, as shown in Figure S2).

We begin by investigating the influence of a circularly confined low-gradient-energy region on ferroelectric domain structure. As illustrated in Figure 1, we define a disk-shaped domain where the reduced second-order gradient coefficient $G_{11}=0.1$, embedded in a matrix with $G_{11}=0.6$. Figure 1a–d show disks of increasing radius, while Figure 1e–h display the corresponding polarization configurations obtained via our Fourier-space perturbation method. Strikingly, within the low-$G_{11}$ region, the system forms fine, fragmented nanodomains, whereas the high-$G_{11}$ exterior stabilizes large, uniform domains. This behavior arises because a smaller gradient coefficient reduces the energetic cost of creating domain walls, thereby promoting domain subdivision to minimize total free energy. Conversely, a larger ${\;G}_{11}$ suppresses polarization rotation, favoring macroscopic alignment. This result demonstrates that gradient energy heterogeneity can serve as a powerful design parameter for nanoscale domain engineering, analogous to strain or composition patterning.

**Figure 2:**
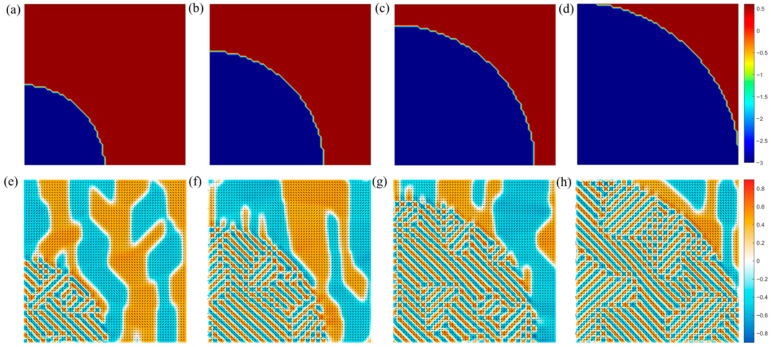
$G_{11}$ distribution and ferroelectric/antiferroelectric domain structures. (**a**–**d**) Circular ferroelectric anisotropy with antiferroelectric regions ($G_{11}=-3.0$) occupying 20%, 40%, 60%, and 80% of the total area, respectively. (**e**–**h**) The corresponding domain structures.

The distinction between FE and AFE ordering hinges critically on the sign of the second-order gradient energy coefficient $G_{11}$. In standard ferroelectric systems, $G_{11}>0$ favors parallel alignment of neighboring polarization vectors, minimizing the gradient energy. In contrast, AFE order emerges when $G_{11}<0$, promoting antiparallel coupling between adjacent dipoles—a configuration stabilized by a positive fourth-order term $H_{11}>0$ that prevents divergence. To model coexisting FE and AFE phases—a scenario highly relevant to relaxor–AFE composites for high-efficiency energy storage—we introduce a spatially varying ${\;G}_{11}$: setting $G_{11}=-3.0$ inside a circular region (AFE core) and $G_{11}=0.6$ in the surrounding matrix (FE shell), as shown in Figure 2a. Figure 2b–d correspond to AFE volume fractions of 20%, 50%, and 80%, respectively. At 20% (Figure 2e), the AFE core exhibits a well-defined 2 × 2 oxygen-octahedral (O-phase) antiferroelectric pattern, characterized by alternating up–down polarization along the [110] direction, while the exterior displays uniform O-phase ferroelectric order. As the AFE fraction increases, the antiparallel domains progressively dominate, with sharp interfaces maintained between FE and AFE regions. These results not only confirm the physical realism of our model but also validate the accuracy and robustness of the Fourier-space perturbation method in capturing complex phase coexistence under strong gradient anisotropy.

**Figure 3:**
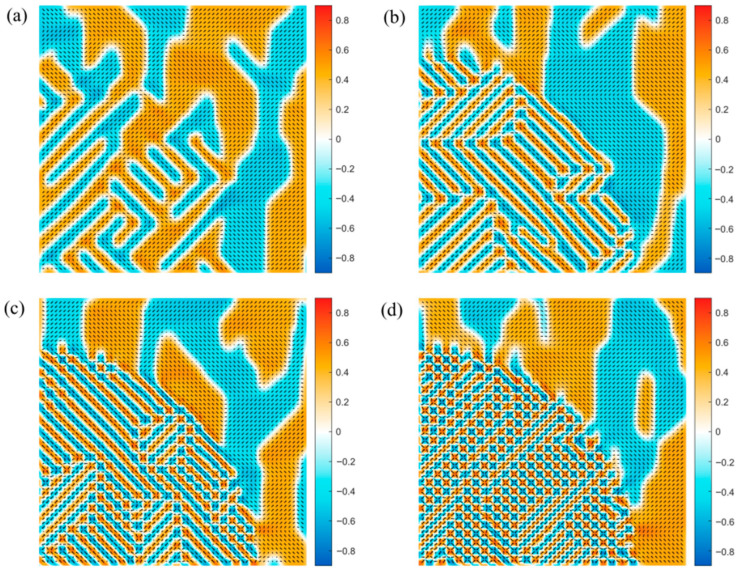
Ferroelectric/antiferroelectric domain structures with different $G_{11}$. (**a**–**d**) Circular antiferroelectric anisotropy with $G_{11}$ of −1.5,−2.5,−3.0 and −3.5.

To further elucidate the role of gradient energy magnitude, we fix the AFE volume fraction and systematically vary $G_{11}$ within the AFE region from −1.5 to −3.5 (Figure 3a–d). At $G_{11}=-1.5$, the AFE period is large (4 × 4), resembling FE domains but with antiparallel alignment—indicating weak antiferroic coupling. As $G_{11}$ decreases to −2.5, the period shortens to 2 × 2, reflecting stronger nearest-neighbor antiparallel interaction. At $G_{11}=-3.0$, the system not only sustains the 2 × 2 AFE order but also nucleates topological vortex-like domains at domain junctions. These emerge because the strong antiparallel coupling overwhelms the long-range influence of higher-order gradient and elastic terms, driving each dipole to minimize local energy by aligning antiparallel to all neighbors—a configuration that naturally leads to closed-loop or vortex textures. When $G_{11}$ reaches −3.5, topological domains become dominant across the entire AFE region. The domain period n (number of lattice sites per modulation period) decreases monotonically with increasing magnitude of $\left|G_{11}\right|$, following a scaling relation: $n\propto \frac{1}{\sqrt{\left|G_{11}\right|}}$. This behavior is consistent with the theoretical prediction from the Landau–Ginzburg framework [24]. This domain evolution aligns quantitatively with prior theoretical predictions based on pure antiferroelectric phase-field models, thereby validating the high precision of our anisotropic gradient energy formulation [5].

**Figure 4:**
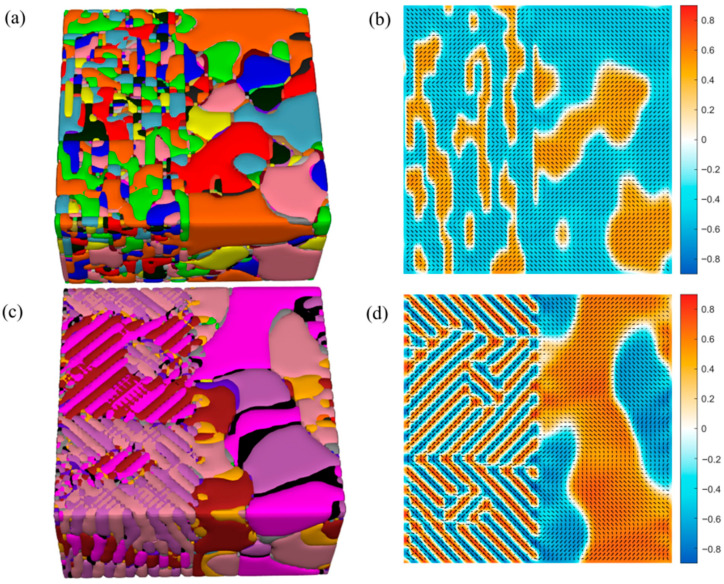
3d ferroelectric/antiferroelectric domain structures with inhomogeneous gradient coefficients. (**a**) The 3d domain structure(Different colors represent different domain structures, as shown in Figure S2) with the left half ($G_{11}=0.1$) and the right half side ($G_{11}=0.6$). (**b**) The polarization vector diagram in the xy cross-section (at z = 16 nm). (**c**) The 3d antiferroelectric domain structure with the left half ($G_{11}=-3.0$) and the right half side ($G_{11}=0.6$). (**d**) The polarization vector diagram in the xy cross-section (at z = 16 nm).

Critically, our method is not limited to two dimensions. We extend the simulation to full 3D, demonstrating its scalability for realistic material architectures. For the 3D simulation, we used a grid of 128Δx × 128Δy × 32Δz with Δx = Δy = Δz = 1.0 nm. A normalized time step Δt = 0.02 was used, with convergence assessed by a residual criterion of 10^−3^, consistent with the 2D cases. Figure 4a shows a 3D FE system with left/right halves assigned $G_{11}\;=\;0.1$ and $G_{11}=0.6$, respectively, using identical LGD potential and material parameters (e.g., PZO-based). The xy-plane slice at z = 16 nm (Figure 4b) clearly reveals domain size modulation across the vertical interface, with finer domains on the left and coarser ones on the right—directly mirroring 2D predictions. More significantly, in the FE-AFE composite (Figure 4c,d), where the left half has $G_{11}=-2.5$ (AFE) and the right half $G_{11}=0.6$ (FE), a sharp transition to 2 × 2 AFE stripe domains is observed throughout the 3D volume. Polarization vector plots confirm the antiparallel periodicity, and the domain walls remain coherent across the z-direction. The excellent agreement between 2D and 3D results confirms that our Fourier-space perturbation method maintains numerical stability, accuracy, and computational efficiency in three dimensions, overcoming a major limitation of conventional real-space solvers.

## 5. Conclusions

In conclusion, this work addresses a critical limitation in the phase-field modeling of ferroelectric/antiferroelectric (FE/AFE) composites by establishing an efficient and accurate perturbation framework for anisotropic gradient energy. Unlike previous Fourier spectral perturbation methods that either assume constant gradient coefficients or treat anisotropy and spatial heterogeneity separately, our formulation provides a unified framework that simultaneously accounts for a full anisotropic gradient energy tensor with arbitrary spatial variations. This capability is achieved by decomposing the tensor into a homogeneous part (handled implicitly) and a fluctuating part (handled explicitly), allowing efficient iterative solution in reciprocal space. The method therefore fills a critical gap in the phase-field modeling of systems where the gradient energy is both anisotropic and locally heterogeneous—such as ferroelectric/antiferroelectric composites with composition gradients or structural modulations. Numerical experiments confirm its robustness, fast convergence, and ability to faithfully reproduce antiferroelectric phase diagrams and AFE-FE transition pathways under various gradient energy conditions. Moreover, the algorithm’s inherent scalability to three dimensions provides direct access to the evolution of complex nanodomain textures and polarization topologies, offering a powerful and extensible tool for exploring high-dimensional anisotropic phenomena in FE/AFE systems.

By bridging the gap between microscopic chemical disorder—such as that arising from compositional variations/random defects (Pb(Zr,Ti)O_3_ system with Ti content x ≤ 0.1 as a concrete case where gradient energy heterogeneity arises from chemical substitution or defect)—and macroscopic electromechanical responses, our approach enables the construction of gradient energy anisotropy in ferroelectric/antiferroelectric systems induced by chemical disorder. This capability provides a powerful computational tool for phase-field simulations of piezoelectric and dielectric energy storage applications, which will be demonstrated in our future work.

## Data Availability

The original contributions presented in this study are included in the article/Supplementary Material. Further inquiries can be directed to the corresponding authors.

## Supplementary Materials

The following supporting information can be downloaded at: https://www.mdpi.com/article/10.3390/ma19071445/s1.
