# Supplementary material for: A Perturbation Model of Gradient Energy Anisotropy for Phase-Field Simulation of Ferroelectrics
Source: Materials (Basel). 2026 Apr 4;19(7):1445. doi: 10.3390/ma19071445 (PMC13074933; doi:10.3390/ma19071445)
Supplement: Supplementary file 1 [file materials-19-01445-s001.zip › materials-4180042-supplementary.pdf]

By performing the variational derivative of the polarization gradient energy, we obtain

$$\begin{aligned}\frac{\delta F_G}{\delta P_1} = & -\frac{\partial G_{11}}{\partial x_1}P_{1,1} - G_{11}(\mathbf{r})\frac{\partial^2 P_1}{\partial x_1^2} \\ & -\frac{\partial G_{12}}{\partial x_1}\left(P_{2,2}+P_{3,3}\right) - G_{12}(\mathbf{r})\left(\frac{\partial^2 P_2}{\partial x_1 \partial x_2}+\frac{\partial^2 P_3}{\partial x_1 \partial x_3}\right) \\ & -\frac{\partial G_{44}}{\partial x_2}\left(P_{1,2}+P_{2,1}\right) - G_{44}(\mathbf{r})\left(\frac{\partial^2 P_1}{\partial x_2^2}+\frac{\partial^2 P_2}{\partial x_1 \partial x_2}\right) \\ & -\frac{\partial G_{44}}{\partial x_3}\left(P_{1,3}+P_{3,1}\right) - G_{44}(\mathbf{r})\left(\frac{\partial^2 P_1}{\partial x_3^2}+\frac{\partial^2 P_3}{\partial x_1 \partial x_3}\right) \\ & -\frac{\partial G'_{44}}{\partial x_2}\left(P_{1,2}-P_{2,1}\right) - G'_{44}(\mathbf{r})\left(\frac{\partial^2 P_1}{\partial x_2^2}-\frac{\partial^2 P_2}{\partial x_1 \partial x_2}\right) \\ & -\frac{\partial G'_{44}}{\partial x_3}\left(P_{1,3}-P_{3,1}\right) - G'_{44}(\mathbf{r})\left(\frac{\partial^2 P_1}{\partial x_3^2}-\frac{\partial^2 P_3}{\partial x_1 \partial x_3}\right)\end{aligned} \tag{S1}$$

To provide further support, we include a theoretical explanation of why the semi-implicit Fourier-space perturbation method offers superior stability and convergence. The gradient energy term in the TDGL equation is:

$$\frac{\partial P}{\partial t} = L\nabla \cdot (G(\mathbf{x})\nabla P) + \cdots \tag{S2}$$

In Fourier space, this becomes a convolution:

$$\mathcal{F}\left[\frac{\partial P}{\partial t}\right] = -L\left[\hat{G}(\mathbf{k}) * \left(-\parallel \mathbf{k} \parallel^2 \hat{P}(\mathbf{k})\right)\right] \tag{S3}$$

By decomposing $G(\mathbf{x}) = \bar{G} + \delta G(\mathbf{x})$, where $\bar{G}$ is the spatial average, we obtain:

$$\nabla \cdot (G\nabla P) = \bar{G}\nabla^2 P + \nabla \cdot (\delta G \nabla P) \tag{S4}$$

A semi-implicit time discretization (implicit for the constant-coefficient part, explicit for the perturbation) yields:

$$\frac{\hat{P}^{n+1} - \hat{P}^{n}}{\Delta t} = -L\bar{G} \parallel \mathbf{k} \parallel^2 \hat{P}^{n+1} - L\mathcal{F}[\nabla \cdot (\delta G \nabla P^n)] \tag{S5}$$

Rearranging:

$$\hat{P}^{n+1} = \frac{\hat{P}^{n} - L\Delta t\, \mathcal{F}[\nabla \cdot (\delta G \nabla P^n)]}{1 + L\Delta t \bar{G} \|\mathbf{k}\|^2} \tag{S6}$$

For the linear constant-coefficient part, the amplification factor is $1/(1 + L\Delta t\bar{G} \parallel \mathbf{k} \parallel^2)$, which is always less than 1. This makes the scheme unconditionally stable with respect to the dominant gradient term, allowing much larger time steps than explicit methods. For the perturbation part, its explicit treatment does not compromise overall stability provided the perturbation magnitude is reasonable. In contrast, fully explicit schemes require a severe time-step restriction:

$$\Delta t \leq \frac{1}{L\max(G(\mathbf{x}))\,\|\mathbf{k}\|_{\max}^2} \tag{S7}$$

which scales as $\Delta x^2/N^2$ and becomes prohibitively small for high-resolution simulations.

The same analysis applies to the fourth-order gradient energy used in antiferroelectric systems, where we decompose $H(\mathbf{x}) = \bar{H} + \delta H(\mathbf{x})$ and obtain:

$$\hat{P}^{n+1} = \frac{\hat{P}^n - L\Delta t\,\mathcal{F}[\nabla^2(\delta H \nabla^2 P^n)]}{1 + L\Delta t \bar{H}\|\mathbf{k}\|^4} \tag{S8}$$

which similarly yields unconditional stability.

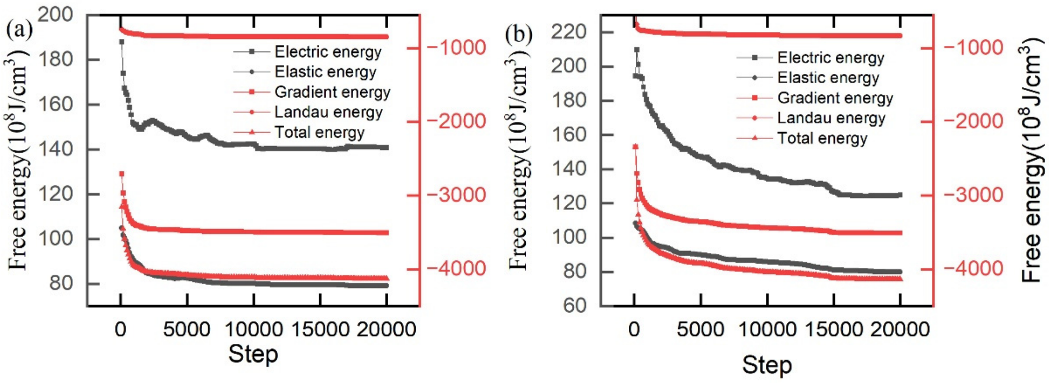

Figure S1. Converge test with different time step of 0.02(a) and 0.1(b).

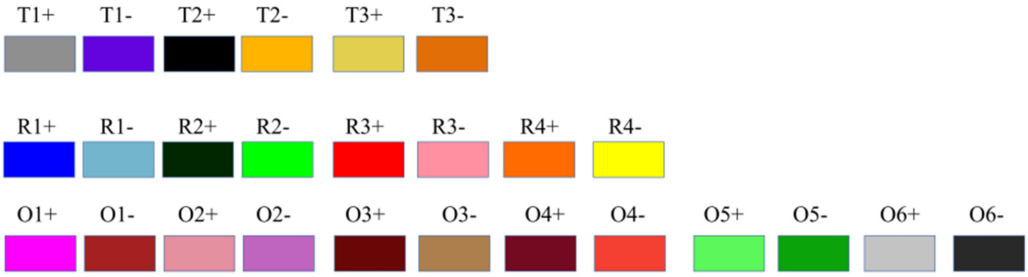

Figure S2. Ferroelectric domains represented by different colors..
